# Spatiotemporal patterns of zoonotic cutaneous leishmaniasis in the high-burden provinces of Morocco, 1995–2022: an exploratory analysis of environmental and socio-demographic correlates

**DOI:** 10.1051/parasite/2026045

**Published:** 2026-08-13

**Authors:** Chaymaa Harkat, Denis Sereno, Oussama Rida, Hicham Hafiane, Souad Bouhout, Laila Hoummadi, Kholoud Kahime

**Affiliations:** 1 Essaouira Higher School of Technology, Interdisciplinary Laboratory for Research in Environment, Management, Energy and Tourism (LIREMET), Cadi Ayyad University (UCA) Marrakech Morocco; 2 IRD, University of Montpellier, UMR177 34032 Montpellier Cedex France; 3 GOINsEcT: Global Infectiology and Entomology Research Group Montpellier Cedex France; 4 Department of Epidemiology and Disease Control, Ministry of Health and Social Protection Morocco; 5 Higher Institute of Nursing Professions and Health Techniques of Marrakesh Morocco; 6 Bio-resources and Food Safety laboratory, Faculty of Sciences and Techniques, Cadi Ayyad University Marrakesh Morocco

**Keywords:** Zoonotic cutaneous leishmaniasis, *Leishmania major*, Environment, Socioeconomic factors, Morocco

## Abstract

*Background*: Zoonotic cutaneous leishmaniasis (ZCL) caused by *Leishmania major* remains a major public health concern in Morocco, especially in certain semi-arid and pre-Saharan regions where ecological conditions favor transmission. In recent decades, the epidemiology of ZCL has evolved significantly, particularly in its temporal patterns and the persistence of transmission in major endemic provinces. In this study, we adopted a retrospective observational approach to explore the long-term spatiotemporal dynamics of ZCL across nine endemic provinces in Morocco and to assess its potential environmental, socio-economic, and demographic determinants using a descriptive analytical framework. *Principal findings*: A total of 58,880 ZCL cases were reported during the study period between 1995 and 2022, with incidence peaks observed in 2010 and 2018, reflecting cyclical epidemic dynamics. The Drâa-Tafilalet region accounted for the highest disease burden, with Zagora province identified as the main epidemiological hotspot. Children aged ≤10 represented the most affected age group (40.8% of total cases), whereas infection rates did not differ significantly between the sexes (female-to-male ratio = 1.17). Higher incidence was observed in hyper-arid provinces characterized by sandy-clay-loam soils. However, incidence was not statistically correlated with either environmental or social variables (*p* > 0.05). *Conclusions*: ZCL remains endemic in Morocco, with persistent hotspots linked to specific ecological contexts. These findings highlight the importance of geographically targeted surveillance and control measures to support the integration of environmental indicators into risk monitoring and early-warning systems for leishmaniasis.

## Abstract (fr)

*Contexte* : La leishmaniose cutanée zoonotique (LCZ), causée par *Leishmania major*, demeure un problème majeur de santé publique au Maroc, en particulier dans certaines régions semi-arides et présahariennes où les conditions écologiques favorisent la transmission. Au cours des dernières décennies, l'épidémiologie de la LCZ a considérablement évolué, notamment en ce qui concerne ses profils temporels et la persistance de la transmission dans les principales provinces d'endémie. Cette étude adopte une approche observationnelle rétrospective pour explorer la dynamique spatio-temporelle à long terme de la LCZ dans neuf provinces marocaines d'endémie et pour évaluer ses déterminants potentiels, environnementaux, socio-économiques et démographiques, à l'aide d'un cadre d'analyse descriptive. *Principaux résultats* : Au total, 58 880 cas de LCZ ont été signalés entre 1995 et 2022, avec des pics d'incidence observés en 2010 et 2018, reflétant une dynamique épidémique cyclique. La région de Drâa-Tafilalet a enregistré la charge de morbidité la plus élevée, la province de Zagora étant identifiée comme le principal foyer épidémiologique. Les enfants âgés de 10 ans ou moins constituaient la tranche d'âge la plus touchée (40,8 % du total des cas), tandis qu'aucune différence significative des taux d'infection n'a été observée entre les sexes (rapport femmes/hommes = 1,17). Une incidence plus élevée a été relevée dans les provinces hyper-arides caractérisées par des sols sablo-argilo-limoneux. Toutefois, aucune corrélation statistiquement significative n'a été établie entre l'incidence et les variables environnementales ou sociales étudiées (*p* > 0,05). *Conclusions* : La LCZ reste endémique au Maroc, avec des foyers persistants liés à des contextes écologiques spécifiques. Ces résultats soulignent l'importance de mesures de surveillance et de lutte géographiquement ciblées, afin de favoriser l'intégration d'indicateurs environnementaux dans les systèmes de suivi des risques et d'alerte précoce concernant la leishmaniose.

## Introduction

Cutaneous leishmaniasis (CL), a vector-borne disease caused by protozoan parasites of the genus *Leishmania* (L.), remains a major public health concern and is endemic in nearly 100 countries worldwide. It is estimated that approximately 12 million people are currently infected [32]. The geographic distribution of CL has expanded in recent decades, particularly in arid and semi-arid regions undergoing environmental change. Consequently, CL continues to represent a serious public health challenge in the Maghreb region, where zoonotic cutaneous leishmaniasis caused by *Leishmania major* remains endemic in Morocco, Algeria, and Tunisia and displays heterogeneous eco-epidemiological patterns [5]. In Morocco, the World Health Organization classifies the country as having a high burden of CL, with an estimated incidence of 5.62 cases per 100,000 inhabitants and approximately 14% of the population living at risk of infection [31].

The zoonotic form of CL is caused by *L. major* and was first reported in southern Morocco in the late 1970s and has since been associated with several outbreaks affecting arid regions along the northern edge of the Sahara Desert, particularly in Ouarzazate and Oujda desert [19, 34]. Transmission is primarily mediated by the sandfly *Phlebotomus papatasi*, the proven vector of this parasite. Historically, outbreaks have shown a wave-like progression from west to east across pre-Saharan regions [33]. Climate change is expected to expand the habitats of the primary vector and reservoir host, which could increase the transmission of ZCL in new regions including eastern, High Atlas, and Rif regions, while its broader potential distribution is across the central-eastern side of Morocco [11]. Disease dynamics have been associated with environmental changes, including climate variability, anthropogenic activities, and socioeconomic factors that influence both vector and reservoir populations [7, 34]. Moreover, ZCL exhibits a cyclic epidemiological pattern characterized by alternating endemic and epidemic phases, with intense outbreaks lasting two to three years followed by inter-epidemic periods exceeding five years, during which susceptibility is particularly high among children and newly arrived populations [26]. Across North Africa, *Leishmania major* transmission is primarily maintained by two major rodent reservoirs, *Psammomys obesus* and *Meriones shawi*, whose relative importance varies geographically according to local ecological conditions. These reservoir systems are associated with different habitat characteristics and may therefore respond differently to environmental drivers of ZCL transmission [5, 22]. Because reservoir-specific information was not available in the surveillance database, the present analysis considered ZCL to be a single epidemiological entity and did not distinguish between transmission systems associated with these two reservoirs. In Morocco, *Meriones shawi* is considered the principal reservoir host in many arid and semi-arid endemic foci [1, 19].

In this study, we focused on ZCL caused by *L. major* since the historical and ecological distribution of ZCL in Morocco is geographically more restricted, and transmission is primarily associated with *Ph. papatasi* and rodent reservoirs mainly in semi-arid and pre-Saharan regions, in contrast to the more northern and central distribution of other cutaneous leishmaniasis forms. Because the spatiotemporal distribution of ZCL in Morocco is strongly influenced by ecological conditions, environmental, socio-economic and demographic factors are likely to contribute to the persistence of the disease in specific endemic foci. Understanding these dynamics is essential for improving surveillance strategies and guiding targeted control measures in endemic hotspots. However, despite the availability of long-term surveillance data, few studies have comprehensively analyzed the spatiotemporal dynamics of ZCL across Morocco while integrating environmental (including the normalized difference vegetation index, bioclimatic stage, soil type and altitude), socioeconomic (poverty, vulnerability, and urbanization) and demographic determinants. In this context, the present study aimed to characterize the spatiotemporal patterns of ZCL in Morocco over a 28-year period (1995–2022). Specifically, we analyzed temporal trends and spatial distribution at multiple administrative scales and explored potential associations between disease incidence and the selected variables.

## Methods

### Ethics considerations

This study used aggregated surveillance data provided by the Moroccan Directorate of Epidemiology and Disease Control (DELM), including the number and geographic distribution of reported cases as well as the age and sex distribution of affected individuals. The dataset did not contain any personally identifiable information. The use of these anonymized surveillance data was conducted in accordance with the institutional policies of the Moroccan Ministry of Health and Social Protection.

### Study area and population

Morocco is administratively divided into 12 regions and 75 provinces. This study focused on the most endemic foci for ZCL: Errachidia, Zagora, Tinghir, Ouarzazate, and Midelt provinces (Drâa-Tafilalet region) in southeastern Morocco; Boulemane province (Fes-Meknes region) in the central part of the country; Jerada and Figuig provinces (Oriental region) in eastern Morocco; and Tata province (Souss-Massa region) in the south.

The altitude of these provinces ranges from 693 to 1690 m above sea level, with Tata representing the lowest elevation and Boulemane the highest. Climatic conditions vary considerably across the study area, extending from hyper-arid and desert climates in the southeastern provinces to more humid bioclimatic stages in the central regions [29]. Vegetation cover also shows strong spatial variability, with sparse vegetation in the southeastern provinces and denser vegetation in Boulemane [30].

Soil types exhibit marked heterogeneity across the study area. Sandy and loam-clay soils dominate in southeastern and southern provinces, whereas Boulemane is mainly characterized by mineral soils. In contrast, eastern provinces are largely dominated by calcareous soils, reflecting a transition from arid environments in the southeast to more humid conditions in eastern and central Morocco [24].

The total population within the study area was approximately 2,194,855 inhabitants according to the 2024 General Population and Housing Census (RGPH). The largest population was recorded in Errachidia province with 425,920 inhabitants. According to RGPH 2024 data, urbanization rates ranged from 17.82% in Zagora to 65.4% in Jerada. Poverty rates also showed marked disparities, ranging from 4.5% in Errachidia to 24.1% in Figuig, while vulnerability rates ranged from 7.6% in Errachidia to 20.3% in Zagora [15].

### Data acquisition and variables

#### Epidemiological data

To assess the evolution of ZCL in Morocco, a retrospective observational study was conducted at both regional and provincial levels covering the period 1995–2022. Epidemiological data were obtained from the Moroccan Directorate of Epidemiology and Disease Control (DELM), which records the number and spatial distribution of reported ZCL cases by sex, age group, and administrative division.

In the Moroccan national leishmaniasis surveillance system, cutaneous leishmaniasis cases occurring in pre-Saharan desert and steppe foci, historically characterized as zoonotic CL, are epidemiologically attributed to *L. major*. This attribution is based on decades of entomological and parasitological investigations showing that these foci are dominated by *L. major* MON-25, transmitted by *Ph. papatasi* with *Meriones shawi* as the main reservoir, and that *L. tropica* and *L. infantum* have not been documented as circulating there. In our study, cases were identified through the routine national surveillance system, which combines passive detection at primary health centers with active case finding during medical campaigns in these known zoonotic CL foci. Species attribution followed the current Moroccan framework for ZCL surveillance: patients presenting with compatible skin lesions (ulcerative or ulceronodular lesions on exposed areas), with parasitological confirmation by positive smear microscopy, and residing in or recently having stayed in a pre-Saharan *L. major*–endemic commune, were classified as *L. major* ZCL. In these specific foci, routine molecular typing is not systematically performed because previous studies have repeatedly confirmed *L. major* as the exclusive or overwhelmingly predominant etiologic agent, and surveillance guidelines therefore allow species attribution based on this stable eco-epidemiological profile [12]. In contrast, in mixed or urban foci where *L. tropica* or *L. infantum* circulate, individual species identification by molecular methods is recommended, but this situation does not apply to the pre-Saharan areas considered in our analysis [4].

Annual incidence rates were calculated per 100,000 inhabitants using corresponding annual population estimates [15].

#### Environmental data

Environmental variables considered in this study included altitude, normalized difference vegetation index (NDVI), soil type, and bioclimatic stage for the selected provinces. Altitude was recorded as the mean elevation of each province based on Google Earth Pro (https://www.google.com/earth/versions/#earth-pro). Vegetation intensity was assessed using the NDVI, derived from Landsat satellite imagery at 30 m spatial resolution provided by the United States Geological Survey (USGS) (https://earthexplorer.usgs.gov/). For each province, the NDVI was calculated annually spanning the period 1995 to 2022 with a focus on April images to ensure seasonal consistency, and were aggregated by averaging annual values to obtain long-term NDVI over the study period. Spatial processing and data extraction were performed using ArcMap 10.8.

Additional environmental information, including soil types, was obtained from the FAO digital soil maps [14]. Data on bioclimatic stages were sourced from the Moroccan Ministry of Territorial Development, Water and Environment [24]. Soil type and bioclimatic stage were assigned at the provincial level according to the dominant class occupying the largest surface area within each province.

#### Socioeconomic and demographic data

Socioeconomic indicators, including poverty, vulnerability, urbanization rates, and demographic characteristics, were obtained from official publications of the Moroccan High Commission for Planning [15]. These indicators are available from national population censuses conducted at decennial intervals. Accordingly, socioeconomic data were aggregated in 10-year periods across the study timeframe to evaluate their potential associations with ZCL incidence. Agricultural activity indicators were not available at a consistent spatial and temporal scale for the entire study period and could therefore not be formally included in the quantitative analyses; however, their potential influence on ZCL transmission is considered qualitatively in the interpretation of the results.

### Data aggregation and statistical analysis

ZCL incidence data are officially reported and publicly accessible primarily at the provincial level. The province was used as the highest spatially comparable unit across datasets, because communal-level surveillance and covariate data were not consistently available for the full study period. This choice allowed for long-term comparative analysis but may mask finer focal heterogeneity in transmission. Although this resulted in a relatively small number of analytical units (*n* = 9 provinces), this administrative level remains epidemiologically relevant because provincial health authorities are responsible for disease surveillance and intervention planning in Morocco. Epidemiological time series were examined for internal consistency at the provincial level, and any obvious data-entry errors or implausible values were cross-checked against original reports and corrected when possible. Years with missing or incomplete provincial records were excluded from temporal analyses or aggregated with adjacent years to maintain coherent time intervals. No statistical imputation of missing incidence values was performed, and all analyses were conducted on the available observed data.

Associations between ZCL incidence and environmental variables (altitude and NDVI) were assessed using Spearman’s rank correlation test to evaluate monotonic relationships. Qualitative environmental variables, including soil type and bioclimatic stage, were analyzed using the non-parametric Kruskal–Wallis test. Non-parametric statistical methods were selected because they are less sensitive to distributional assumptions and are more appropriate for analyses involving a limited number of spatial units.

To assess the relationships between ZCL incidence and social indicators (urbanization rate, poverty rate, vulnerability rate, and demographic characteristics), a decennial multilevel analysis was performed based on national population census periods (1995–2004, 2005–2014, and 2015–2024). As socioeconomic data are collected through national censuses at decennial intervals, annual indicators were not available. Therefore, ZCL incidence was aggregated over the corresponding intercensal periods (each 10 years) beginning from 1995 to ensure temporal consistency with socioeconomic variables. This approach allowed harmonization between datasets with differing temporal resolutions, although it masks short-term variability.

To further investigate the relationship between ZCL incidence and human-related factors, a multistep analytical approach was applied. First, univariate Spearman correlation analyses were performed. Second, multivariate ordinary least squares (OLS) regression was conducted using log-transformed incidence rates (log (*x* + 1)) and standardized socioeconomic predictors (*Z*-scores). Third, hierarchical agglomerative clustering using Ward’s method was implemented to identify groups of provinces with similar socio-epidemiological profiles. OLS regression as well as hierarchical clustering were performed using a restricted set of predictors to investigate relative strength of associations. Due to the limited data size, the analyses are interpreted cautiously and regarded as exploratory, as the objective is not to construct a predictive index but to provide a descriptive multivariate profile of provinces. Therefore, all variables were standardized (*z*-scores) to ensure comparability across different scales and no weighting scheme was applied.

In addition, a normalized multi-criteria risk profiling approach integrating epidemiological, environmental, socioeconomic, and demographic indicators was developed to identify provinces with the highest potential transmission risk. The composite risk profile included epidemiological (mean incidence), environmental (altitude, NDVI, soil type, and bioclimatic stage), and socioeconomic/demographic indicators (poverty, vulnerability, urbanization, and demographic variables). Continuous indicators were normalized to a 0–1 scale using min–max transformation. Qualitative variables were converted into ordinal scores reflecting their relative ecological suitability for ZCL transmission based on the literature. In the absence of previous studies providing evidence for differential weighting, equal weighting was applied to avoid introducing subjective bias. Provincial composite profiles were visualized using radar charts.

Statistical significance was defined at *p* < 0.05 with a 95% confidence level. All data processing, standardization, and statistical analyses were conducted using Python software.

## Results

### Spatiotemporal and demographic trends of ZCL in Morocco (1995–2022)

#### Temporal trends

**Figure 1:**
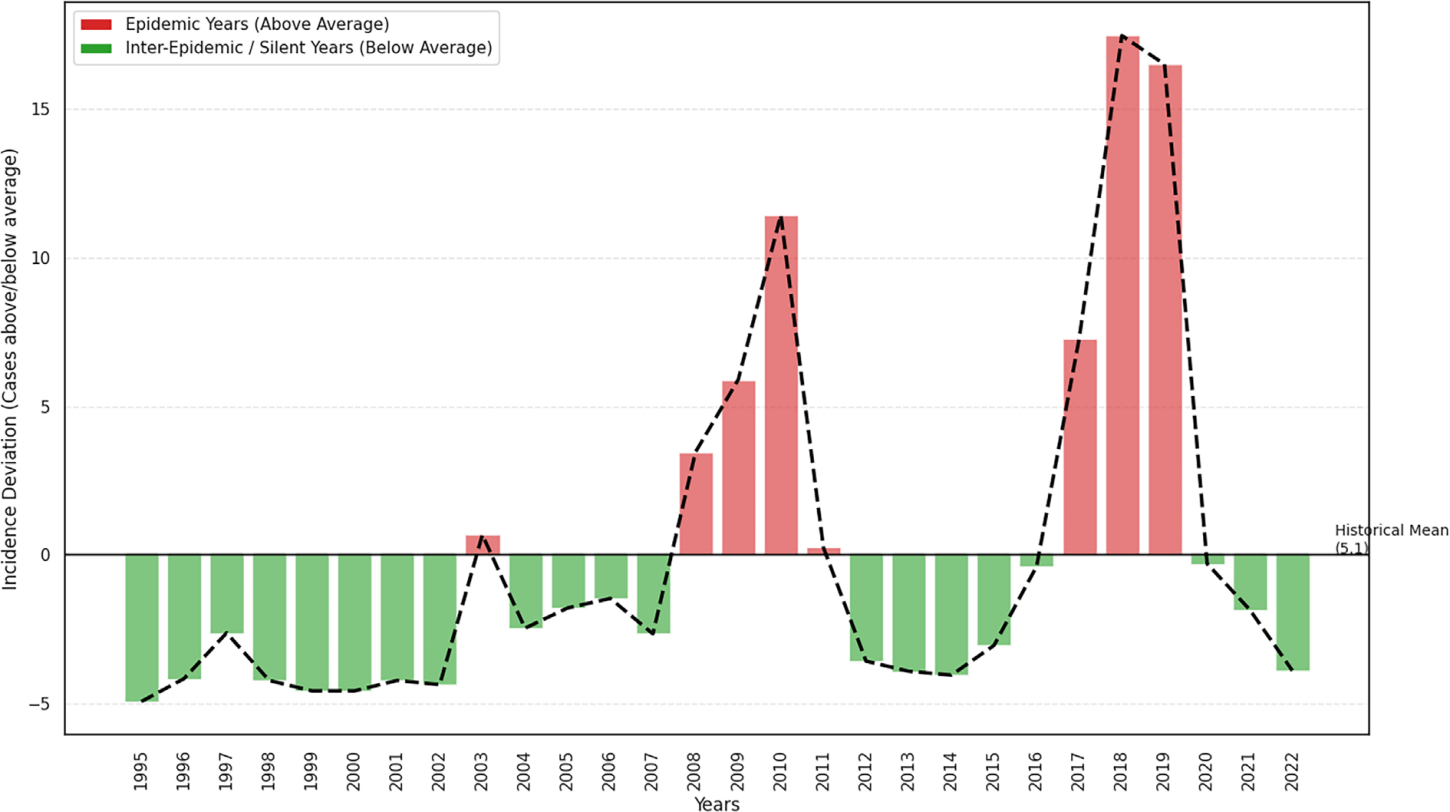
Cyclic patterns of zoonotic cutaneous leishmaniasis in Morocco based on annual deviations from the national mean incidence during the period 1995–2022. The horizontal reference line represents the long-term national mean incidence (5.1 cases per 100,000 inhabitants).

A total of 58,880 ZCL cases were reported in Morocco between 1995 and 2022, corresponding to an average incidence of 7.36 cases per 100,000 inhabitants. Incidence peaked in 2010 (17.5 cases per 100,000) and again in 2018 (24.17 cases per 100,000). Two major epidemic periods were identified: 2008–2011 and 2017–2019. During these periods, incidence levels were markedly higher than the long-term average, whereas lower levels were observed during inter-epidemic years, indicating a cyclical pattern of ZCL occurrence in this endemic focus (Fig. 1).

#### Spatial trends

**Figure 2:**
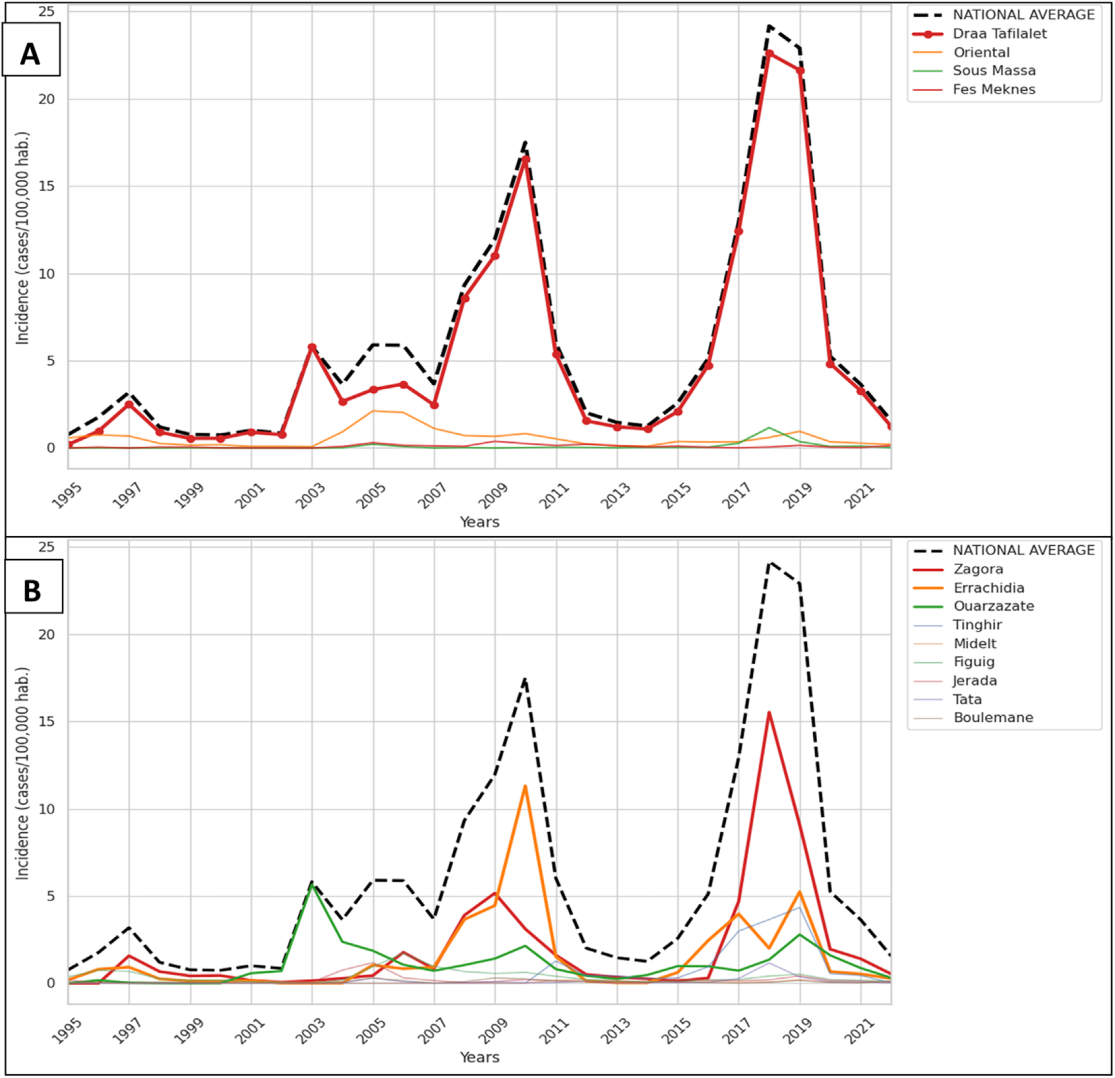
Multi-scale spatiotemporal dynamics of zoonotic cutaneous leishmaniasis in Morocco between 1995 and 2022. (A) Temporal evolution of ZCL incidence at the national and regional scales. (B) Temporal evolution of ZCL incidence at the national and provincial scales.

**Figure 3:**
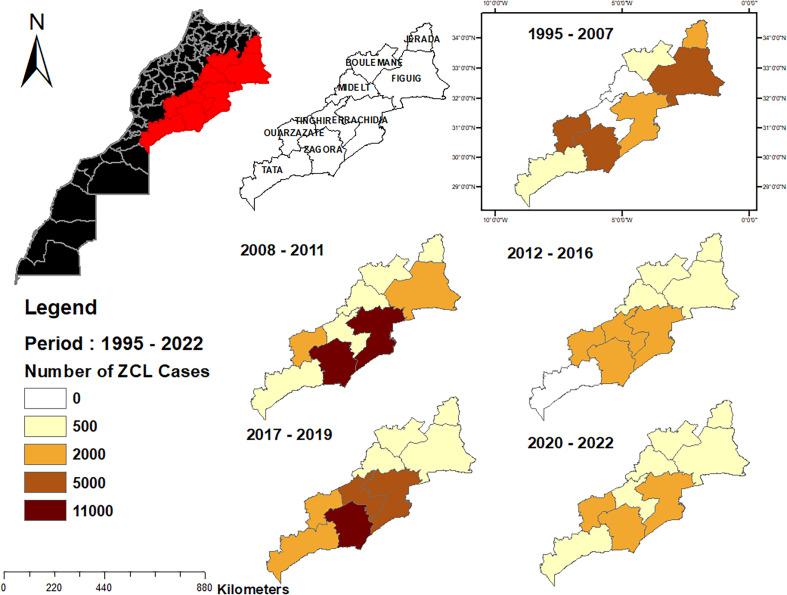
Spatiotemporal distribution of zoonotic cutaneous leishmaniasis cases in Morocco across four epidemiological periods between 1995 and 2022.

The Drâa-Tafilalet region showed the highest ZCL burden compared with other regions of Morocco. Within this region, incidence rates varied considerably across provinces. The highest incidence was observed in Zagora (51.94 cases per 100,000 inhabitants), followed by Errachidia (39.7 cases per 100,000), Ouarzazate (27.71 cases per 100,000), and Tinghir (15.57 cases per 100,000). These spatial patterns indicate a heterogeneous distribution of ZCL within the region, with several persistent transmission foci. Moreover, temporal trends observed in Zagora province closely mirrored both regional and national incidence patterns during the study period (Figs. 2 and 3).

#### Demographic characteristics

**Figure 4:**
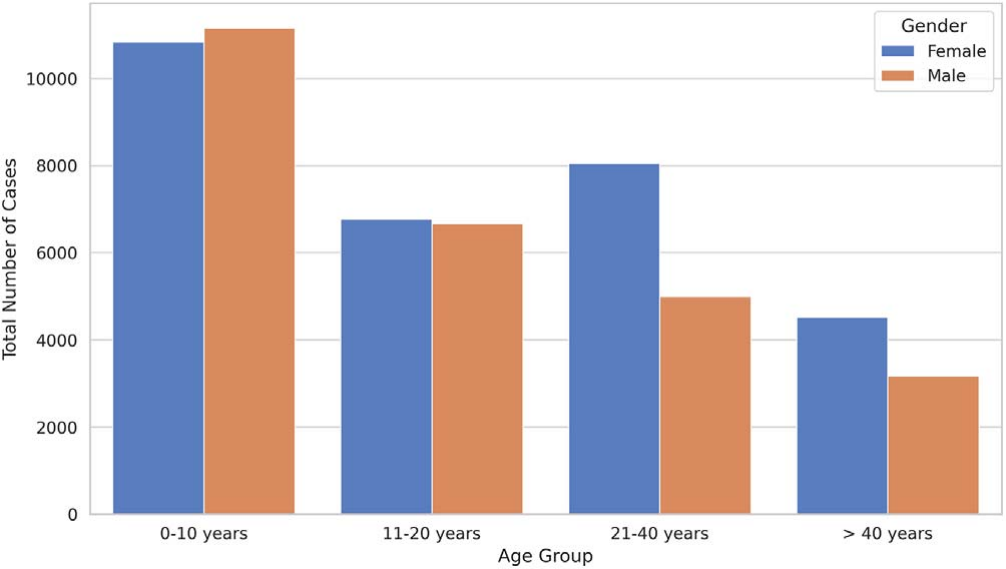
Demographic characteristics of zoonotic cutaneous leishmaniasis cases in Morocco. Distribution of reported cases by age group and sex during the study period (1995–2022).

Children aged ≤10 years represented the most affected age group, accounting for 21,995 cases (40.8%). Infection rates were similar between sexes, with 46.3% of cases occurring in males and 53.7% in females (female-to-male ratio = 1.17), indicating no substantial sex-related difference in disease occurrence (Fig. 4).

### Assessment of the impact of key environmental correlates on ZCL incidence at the provincial level

**Figure 5:**
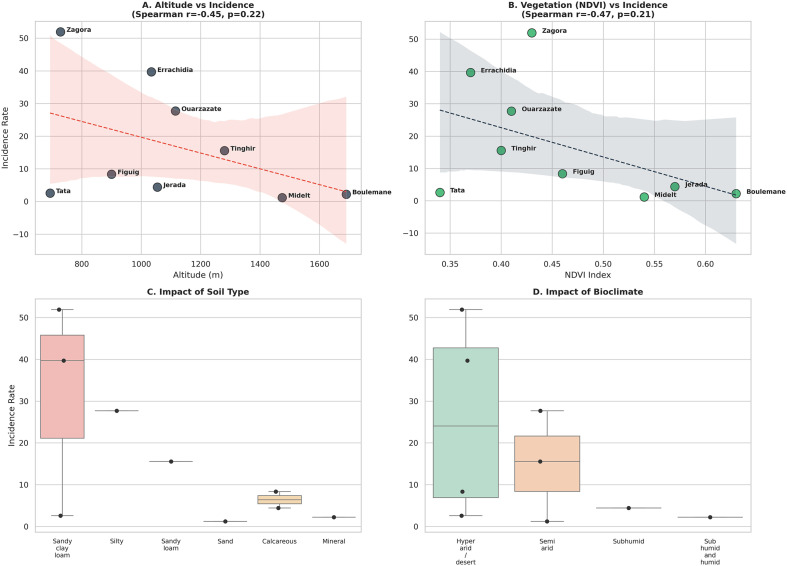
Associations between environmental determinants and zoonotic cutaneous leishmaniasis incidence at the provincial scale. (A) Relationship between incidence and altitude. (B) Relationship between incidence and vegetation cover measured by the normalized difference vegetation index (NDVI) (scatter plots). (C) Distribution of incidence according to soil type. (D) Distribution of incidence according to bioclimatic stage (boxplots).

To explore environmental heterogeneity, mean ZCL incidence was descriptively examined in relation to four environmental parameters: altitude, NDVI, bioclimatic stage, and soil type across the selected provinces. All analyses are presented for an exploratory framework (Fig. 5).

(A) *Altitude*: Zagora province, located at a relatively low altitude (728 m), showed the highest ZCL incidence rate, whereas Boulemane province, located at a higher altitude (1690 m), showed the lowest incidence. However, other low-altitude provinces, such as Tata province, exhibited relatively low incidence rates, resulting in a negative and non-significant correlation between altitude and ZCL incidence (*r* = −0.45, *p* = 0.22).
(B) *NDVI*: Provinces characterized by low NDVI values, such as Zagora, tended to exhibit higher incidence rates, whereas provinces with higher NDVI values, such as Boulemane, showed lower incidence levels. Yet, this pattern was not consistent across all endemic provinces. Nevertheless, the correlation between NDVI and ZCL incidence was not statistically significant (*r* = −0.47, *p* = 0.21).
(C) *Soil type*: Marked heterogeneity was observed among soil categories. Provinces characterized by sandy-clay-loam soils showed the highest median incidence rates and the greatest variability. In contrast, provinces dominated by mineral, calcareous, or sandy soils exhibited lower and more homogeneous incidence levels. However, the Kruskal–Wallis test did not show statistical significance (*p* = 0.39), given the restricted number of observations per category. Accordingly, no inference conclusions are taken into consideration.
(D) *Bioclimatic stages*: Incidence levels varied across bioclimatic zones, with significant incidence rates observed in hyper-arid and desert environments, including provinces such as Zagora, Errachidia, Figuig, and Tata. Boxplot distributions indicated substantial variability within these climatic categories. However, no statistical significance was detected (*p* = 0.52).

Overall, the analyses highlight spatial heterogeneity in ZCL incidence across provinces. Furthermore, statistical analyses did not reveal significant correlations between the selected environmental variables and ZCL incidence, likely reflecting the limited number of spatial units included in the analysis (*n* = 9 provinces). However, spatial patterns observed across provinces consistently suggest that ZCL transmission tends to occur in low-altitude and hyper-arid conditions characterized by low vegetation cover and specific soil conditions that may favor both sandfly vectors and rodent reservoirs.

### Correlation of ZCL incidence with key socioeconomic and demographic variables at the provincial level

**Figure 6:**
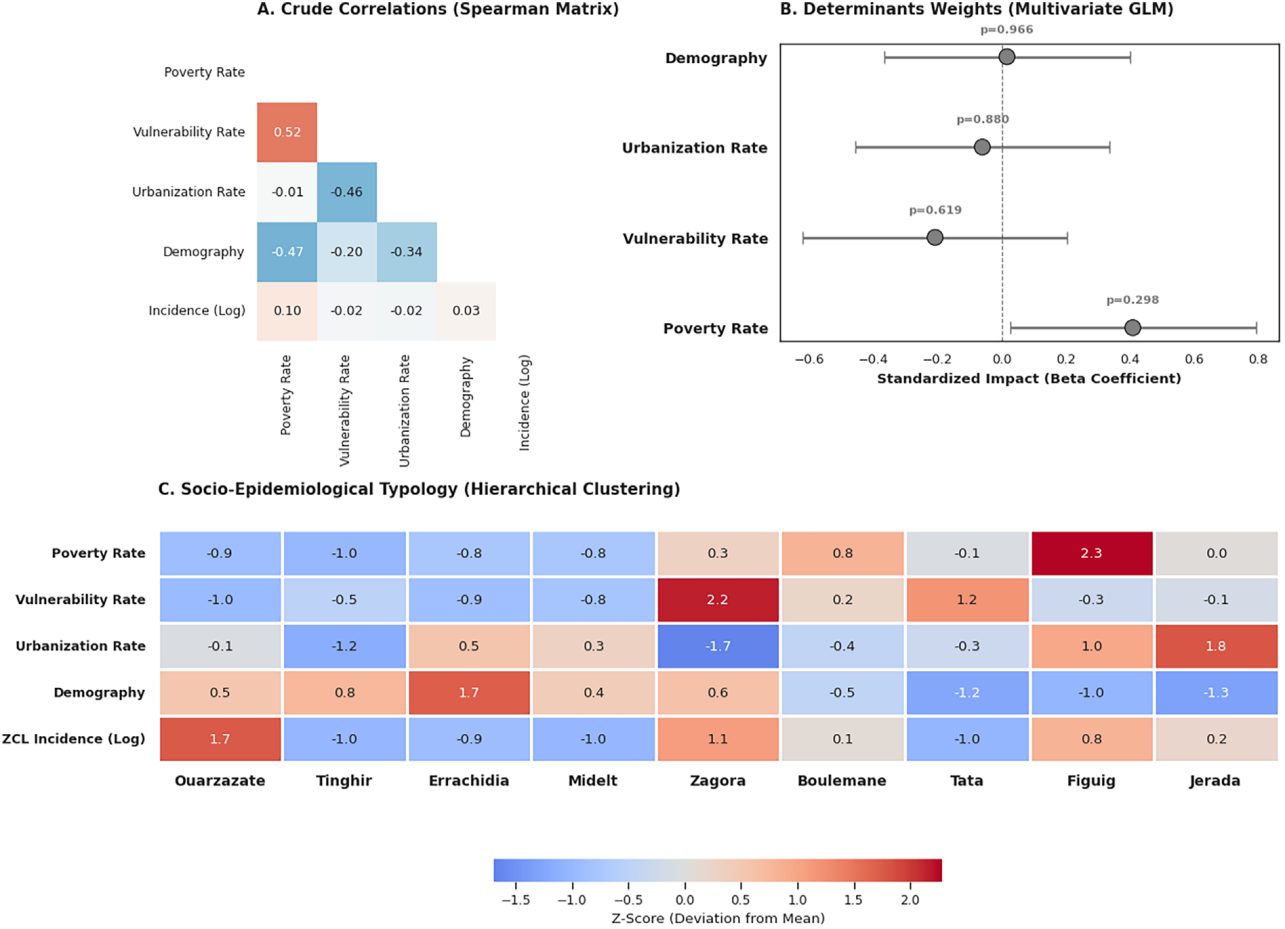
Integrated analysis of socio-demographic determinants of zoonotic cutaneous leishmaniasis. (A) Pairwise Spearman correlation matrix between socio-demographic indicators and log-transformed ZCL incidence. (B) Standardized effects of socio-demographic determinants on ZCL incidence estimated using multivariate ordinary least squares (OLS) regression. (C) Hierarchical clustering–based socio-epidemiological typology of provinces according to demographic, socioeconomic, and epidemiological profiles.

Spearman correlation analyses revealed non-significant associations between log-transformed ZCL incidence and the selected social indicators. Similarly, ordinary least squares (OLS) regression analyses showed that none of the socioeconomic variables significantly predicted ZCL incidence across the study area, as all predictors were associated with high *p*-values (*p* > 0.05). Moreover, hierarchical clustering based on standardized *Z*-scores was subsequently applied to provide a descriptive classification of provinces with similar socio-epidemiological profiles. The analysis revealed heterogeneous patterns among provinces, with no consistent alignment between socioeconomic conditions and ZCL incidence levels. For instance, Ouarzazate exhibited high ZCL incidence despite relatively low poverty and vulnerability levels. Figuig province showed moderate incidence despite having the highest poverty rate. In contrast, Zagora province displayed both elevated vulnerability and relatively high incidence. Given the limited data size and aggregated nature of the data, these analyses are interpreted as exploratory (Fig. 6).

To further investigate the lack of a significant association, a qualitative comparison was conducted between the provinces of Zagora and Tata, based on Hierarchical clustering (Fig. 6C). The two provinces share nearly identical macro-environmental profiles (hyper-arid bioclimate, nearly similar altitude, and similar Sandy_clay_loam soil type). Most importantly, they also share structural socioeconomic vulnerabilities. As shown by their standardized *Z*-scores, both provinces are predominantly rural (*Z*-score = −1.7 for Zagora and −0.3 for Tata) and both exhibit high socio-demographic vulnerability (*Z*-score = 2.2 and 1.2, respectively). Despite sharing this rural vulnerability and similar macroclimates, their epidemiological profiles are significantly opposed: Zagora is a hyper-endemic hotspot (*Z*-score = 1.1), while Tata is a nearly silent zone (*Z*-score = −1.0). Consequently, vegetation density appears to be a distinguishing factor, with Zagora having a notably higher NDVI (0.43) compared to Tata (0.34). This higher NDVI may reflect denser oasis agricultural microhabitats in Zagora, which are essential for maintaining the reservoir of *Meriones shawi* rodents.

Overall, these findings indicate considerable spatial heterogeneity and suggest a complex relationship between socioeconomic indicators and ZCL incidence and are not directly observable, with no consistent association observed at the aggregated provincial scale. Moreover, to trigger ZCL transmission requires specific microenvironmental conditions to stimulate disease vectors and reservoirs. Therefore, the composite risk profile developed in this study is intended as a descriptive tool to summarize multidimensional characteristics rather than to infer causal relationships.

### Comparative multivariate characterization of endemic provinces based on standardized determinant domains associated with ZCL transmission

**Figure 7:**
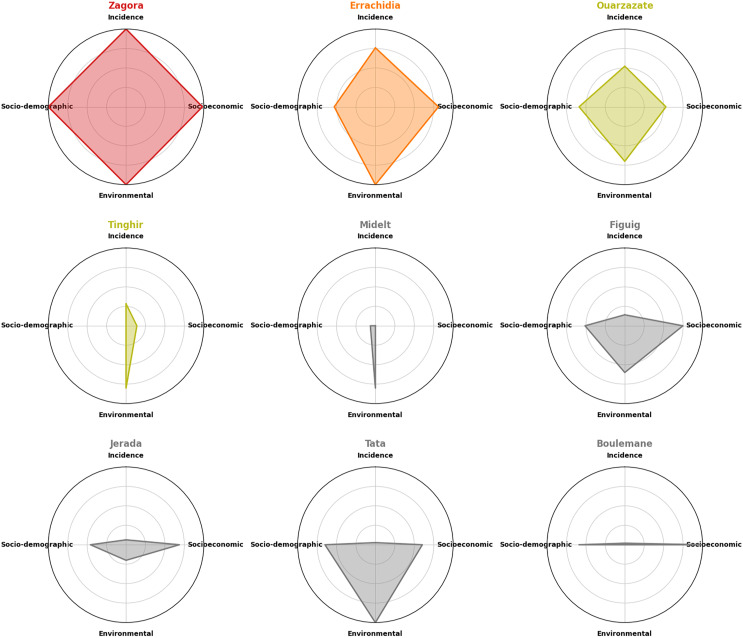
Provincial radar chart profiles illustrating zoonotic cutaneous leishmaniasis incidence and associated socioeconomic, socio-demographic, and environmental determinants derived from normalized composite indicators.

A normalized multi-criteria synthesis was developed to identify the province most representative for environmental monitoring of ZCL transmission. The results (Fig. 7) indicate that Zagora province exhibits the highest composite risk profile when considering epidemiological, ecological, socioeconomic, and demographic indicators. This may reflect the co-occurrence of relatively higher incidence rates, elevated vulnerability indicators and environmental conditions characteristic of hyper-arid bioclimatic zones and sandy-clay-loam soils among this province.

Within this descriptive framework, Zagora appears as the most representative province for integrated environmental monitoring of ZCL dynamics within the studied area. In contrast, provinces such as Midelt and Boulemane showed substantially lower composite profiles associated with differing climatic and environmental contexts.

However, this composite score is entirely descriptive, based on standardized variables (*z*-scores) with equal weighting, and does not rely on any inferential statistical framework. Therefore, this synthesis should not be interpreted as a predictive or causal risk index, but rather as an exploratory tool to summarize multidimensional patterns across provinces.

## Discussion

This study reveals marked spatiotemporal heterogeneity and recurrent epidemic cycles of ZCL across the endemic provinces over nearly three decades. The analytical framework was intended as an exploratory assessment of long-term epidemiological patterns and potential ecological correlates, rather than a causal model of transmission. One of the main findings is the absence of significant statistical associations between ZCL incidence and the selected variables. However, the absence of statistically significant associations with environmental variables should not be interpreted as evidence of a lack of environmental influence. Rather, it likely reflects the limited spatial resolution of the analysis and the relatively small number of spatial units considered. Overall, these findings are consistent with the zoonotic nature of ZCL transmission and support the classification of the selected provinces as a major endemic focus of *L. major*.

The temporal patterns identified in this study highlight marked epidemic cycles, with major incidence peaks observed in 2010 and 2018. These outbreaks were particularly pronounced in Zagora province, which appears to play a central role in shaping the regional epidemiological profile of the disease. Such declines in incidence (2012–2016; 2020–2022) may reflect the development of acquired immunity in exposed populations, mediated by cell-mediated immune responses following infection, which can contribute to a temporary reduction in transmission after epidemic peaks [8]. Conversely, the re-emergence of the disease (2008–2011; 2017–2019) may be facilitated by a progressive decline in population immunity, primarily driven by demographic turnover and the influx of non-immune individuals as a result, multi-year cycles of ZCL incidence are frequently observed, reflecting a dynamic balance between the build-up of herd immunity and the renewal of susceptible populations [9]. The decline in incidence observed after 2019 may partly reflect disruptions in surveillance activities during the COVID-19 pandemic, which affected the detection and reporting of several neglected tropical diseases [28, 36].

Historically, ZCL outbreaks in Morocco have been reported to progress in wave-like patterns from west to east across arid and semi-arid regions [33]. At the national scale, the spatial expansion of CL has been described along pre-Saharan corridors located south of the Atlas Mountains, particularly in the Souss-Massa–Drâa valley and Errachidia province [3]. In eastern Morocco, *L. major* accounted for approximately 89.45% of reported cases between 2016 and 2021, with the highest incidence observed in Figuig province in 2018, particularly among preschool-aged children [27]. Similarly, molecular investigations conducted in Boulemane province confirmed the presence of several (L.) species, with *L. major* representing the predominant parasite [16].

Similar spatiotemporal patterns have been reported in neighboring Algeria, where ZCL caused by *L. major* remains highly endemic in arid and pre-Saharan regions [6]. A 34-year retrospective study conducted in the province of M’Sila revealed recurrent epidemic cycles characterized by alternating endemic and epidemic phases, closely resembling those observed in Morocco. In Algeria, the disease has progressively expanded beyond its historical foci, highlighting the dynamic nature of ZCL transmission across North Africa. Similar to Morocco, transmission is maintained by *Phlebotomus papatasi* and the rodent reservoirs *Psammomys obesus* and *Meriones shawi*, whose population dynamics are strongly influenced by environmental conditions. The Algerian study further suggested that increases in minimum temperatures may contribute to higher endemicity and epidemic outbreaks. In addition, the predominance of childhood infections and the cyclical recurrence of epidemics support the hypothesis that fluctuations in population immunity, combined with the continuous renewal of susceptible individuals, play an important role in shaping ZCL dynamics throughout the Maghreb region. Following periods of intense transmission, naturally acquired immunity may temporarily reduce the number of susceptible individuals and contribute to a decline in incidence before a new susceptible population accumulates through births and migration. These similarities suggest that common ecological and epidemiological drivers may operate across North African endemic foci despite local environmental differences [6, 9].

The trophic cascade leading to a higher incidence of ZCL may have been started by climate variation. Environmental changes affecting vegetation cover and soil composition may influence the spatial distribution of vectors and reservoirs [12, 18]. Changes in temperature can affect sandfly survival and seasonal activity, while precipitation and humidity influence vegetation growth, soil moisture, and the availability of suitable habitats for both vectors and rodent reservoirs, and therefore, the transmission of ZCL. Increasing minimum temperatures that foster endemicity have contributed to a rise in ZCL cases as well [7]. In addition, environmental conditions affecting the population dynamics of rodent reservoirs may play an important role in shaping the temporal patterns of ZCL, such as *Meriones shawi* and *Psammomys obesus* populations, and contribute to sustaining the circulation of *L. major*, particularly in the pre-Saharan regions of Morocco [22]. In arid and semi-arid environments, such as southeastern Morocco, even small variations in climatic conditions may lead to substantial changes in vector abundance and reservoir population dynamics. Although climatic variables were not directly included in the present analysis, their influence likely contributes to the temporal fluctuations observed in ZCL incidence. Future studies integrating climatic time-series data with epidemiological surveillance would therefore provide valuable insights into the environmental drivers of ZCL transmission and help improve predictive models and early warning systems.

In Errachidia province, ZCL incidence has been shown to correlate with precipitation patterns and vegetation indices, while minimum temperature appears to influence seasonal transmission dynamics [7]. For example, dam construction can alter soil humidity and vegetation patterns, potentially influencing sandfly density and distribution [13]. Similarly, in Ouarzazate province, ZCL cases have frequently been recorded near oases and irrigated agricultural areas, suggesting that water resources and irrigation systems may create favorable habitats for sandflies [23]. In the eastern region of Morocco, most reported cases (89.5%) occurred in rural settings (86%), where environmental conditions such as large affected surface areas, deforestation, and arid climatic conditions may influence the density and abundance of both sandfly vectors and rodent reservoirs [4, 27].

At the same time, anthropogenic factors such as land-use change, urbanization, migration, and limited access to healthcare may contribute indirectly to the burden of leishmaniasis in vulnerable populations. Environmental change may also facilitate the expansion of sandflies to higher altitudes, potentially increasing disease risk in previously unaffected areas [10, 19, 20]. Socioeconomic conditions may further influence local transmission dynamics, as illustrated by the reported impact of poverty on the spatial expansion of CL in Boulemane province [16].

Consequently, the emergence and re-emergence of neglected tropical diseases such as CL are increasingly associated with environmental changes, ecological disruption, and evolving host–vector interactions [17, 35]. In this context, the development of early warning systems integrating climatic, ecological, and epidemiological data may play a crucial role in anticipating outbreaks and improving disease control. Combining routine surveillance with environmental monitoring, vector and reservoir population assessments, and spatial forecasting models could provide valuable insights for predicting areas at risk of leishmaniasis transmission [2, 21]. Integrated surveillance combining human, animal, and environmental data may improve outbreak anticipation, and such approaches are consistent with the One Health framework [10, 25].

This study has several limitations. First, the analysis was conducted at the provincial level and included a relatively small number of spatial units (*n* = 9), which may limit the statistical power of correlation analyses. Because the number of spatial units included in the statistical analyses was limited, the results should be interpreted as exploratory rather than causal. In addition, surveillance data prior to 2009 were available only in aggregated form, and socioeconomic indicators were derived from decennial census data, potentially creating temporal mismatches with annual incidence data. Analyses conducted at finer spatial scales, such as the communal level, would allow more robust statistical inference and better characterization of local transmission heterogeneity. Nevertheless, the provincial scale remains relevant for public health decision-making, as it corresponds to the administrative level at which disease control programs are implemented. The main contribution of this study is therefore not the identification of statistically significant determinants, but rather the characterization of long-term spatiotemporal heterogeneity and the identification of persistent epidemiological hotspots suitable for targeted surveillance. In particular, the recurrent high incidence observed in provinces such as Zagora highlights the value of long-term epidemiological monitoring for guiding control strategies and prioritizing surveillance efforts in endemic areas.

Finally, climatic variables such as temperature, precipitation, wind speed, and humidity, recognized as key determinants of sandfly ecology and leishmaniasis transmission, were not directly incorporated into the present analysis. These climatic factors exhibit strong seasonal and interannual variability and require specific modeling frameworks to capture their dynamic effects. Future studies integrating climatic time-series data with epidemiological surveillance are therefore needed to better understand the climate–ZCL relationship in Morocco. Nevertheless, the present study provides valuable insights into the long-term structural determinants associated with persistent ZCL foci and contributes to improving risk-based public health planning.

## Conclusion

ZCL caused by *L. major* remains endemic in Morocco and shows clear spatiotemporal variability. Our results highlight persistent transmission hotspots, particularly in the Drâa-Tafilalet region, with Zagora province emerging as a major focus. Although the environmental and socio-economic variables examined showed limited statistical associations with incidence, the spatial patterns observed suggest that local ecological conditions likely contribute to disease persistence. Future studies integrating climatic variables and finer spatial analyses are needed to better identify the environmental drivers of ZCL transmission. Strengthening surveillance in high-risk areas will be essential for improving prevention and control strategies. Beyond improving our understanding of long-term epidemiological patterns, these findings highlight the need to integrate epidemiological surveillance with environmental and climatic monitoring in order to develop early warning systems capable of anticipating ZCL outbreaks in endemic regions.

## References

[1] Akhoundi M, Kuhls K, Cannet A, Votýpka J, Marty P, Delaunay P, Sereno D. 2016. A historical overview of the classification, evolution, and dispersion of *Leishmania* parasites and sandflies. PLoS Neglected Tropical Diseases, 10, e0004349. 26937644 10.1371/journal.pntd.0004349 PMC4777430

[2] Al-Koleeby Z, Aboudi AE, Faraj C. 2021. Seasonal changes of sandflies: A study of the endemic outbreak of *Leishmania major* in Zagora, Southeast Morocco. E3S Web of Conferences, 234, 00104.

[3] Alvar J, Vélez ID, Bern C, Herrero M, Desjeux P, Cano J, Jannin J, Boer M den, WHO Leishmaniasis Control Team. 2012. Leishmaniasis worldwide and global estimates of its incidence. PloS One, 7, e35671. 22693548 10.1371/journal.pone.0035671 PMC3365071

[4] Amahmid O, El Guamri Y, Zenjari K, Bouhout S, Ait Moh M, Boraam F, Ait Melloul A, Benfaida H, Bouhoum K, Belghyti D. 2021. Epidemiological features of cutaneous leishmaniasis in diagnosed patients from an endemic area (central Morocco). Journal of Parasitic Diseases, 45, 762–768. 34475658 10.1007/s12639-021-01357-2 PMC8368813

[5] Aoun K, Bouratbine A. 2014. Cutaneous leishmaniasis in North Africa: a review. Parasite, 21, 14. 24626301 10.1051/parasite/2014014 PMC3952656

[6] Benikhlef R, Aoun K, Boudrissa A, Abid MB, Cherif K, Aissi W, Benrekta S, Boubidi SC, Späth GF, Bouratbine A, Sereno D, Harrat Z. 2021. Cutaneous leishmaniasis in Algeria; Highlight on the focus of M’Sila. Microorganisms, 9, 962. 33947003 10.3390/microorganisms9050962 PMC8146893

[7] Bounoua L, Kahime K, Houti L, Blakey T, Ebi KL, Zhang P, Imhoff ML, Thome KJ, Dudek C, Sahabi SA, Messouli M, Makhlouf B, El Laamrani A, Boumezzough A. 2013. Linking climate to incidence of zoonotic cutaneous leishmaniasis (*L. major*) in pre-Saharan North Africa. International Journal of Environmental Research and Public Health, 10, 3172–3191. 23912199 10.3390/ijerph10083172 PMC3774431

[8] Boussoffara T, Chelif S, Ben Ahmed M, Mokni M, Ben Salah A, Dellagi K, Louzir H. 2018. Immunity against *Leishmania major* infection: Parasite-specific granzyme B Induction as a correlate of protection. Frontiers in Cellular and Infection Microbiology, 8, 397. 30483482 10.3389/fcimb.2018.00397 PMC6243638

[9] Chamakh-Ayari R, Chenik M, Chakroun AS, Bahi-Jaber N, Aoun K, Meddeb-Garnaoui A. 2017. *Leishmania major* large RAB GTPase is highly immunogenic in individuals immune to cutaneous and visceral leishmaniasis. Parasites & Vectors, 10, 185. 28416006 10.1186/s13071-017-2127-3 PMC5393016

[10] Cosma C, Maia C, Khan N, Infantino M, Del Riccio M. 2024. Leishmaniasis in humans and animals: A One Health approach for surveillance, prevention and control in a changing world. Tropical Medicine and Infectious Disease, 9, 258. 39591264 10.3390/tropicalmed9110258 PMC11598728

[11] Daoudi M, Outammassine A, Olivier D, Amane M, Beaulieu M, Akarid A, Ndao M, Hafidi M, Boussaa S, Boumezzough A. 2025. Modeling the impact of climate change for the potential distribution of the main vector and reservoirs of zoonotic cutaneous leishmaniasis due to *Leishmania major* in Morocco. Frontiers in Tropical Diseases, 6, 1629454.

[12] El Hamouchi A, Daoui O, Ait Kbaich M, Mhaidi I, El Kacem S, Guizani I, Sarih M, Lemrani M. 2019. Epidemiological features of a recent zoonotic cutaneous leishmaniasis outbreak in Zagora province, southern Morocco. PLoS Neglected Tropical Diseases, 13, e0007321. 30964864 10.1371/journal.pntd.0007321 PMC6474635

[13] El Miri H, Faraj C, Himmi O, Hmamouch A, Maniar S, Laaroussi T, Rhajaoui M, Sebti F, Benhoussa A. 2016. Cutaneous leishmaniasis in Ouazzane and Sidi Kacem provinces, Morocco (1997-2012). Bulletin de la Société de Pathologie Exotique (1990), 109, 376–380. 10.1007/s13149-016-0522-1 27646962

[14] FAO. 2006. World reference base for soil resources 2006: A framework for international classification, correlation and communication. FAO Rome.

[15] HCP. 2025. Site institutionnel du Haut-Commissariat au Plan du Royaume du Maroc – Publications HCP. HCP.

[16] Hmamouch A, El Alem MM, Hakkour M, Amarir F, Daghbach H, Habbari K, Fellah H, Bekhti K, Sebti F. 2017. Circulating species of *Leishmania* at microclimate area of Boulemane Province, Morocco: Impact of environmental and human factors. Parasites & Vectors, 10, 100. 28228154 10.1186/s13071-017-2032-9 PMC5322673

[17] Kahime K, Bounoua L, Sereno D, El Hidan M, Messouli M. 2020. Emerging and re-emerging leishmaniases in the Mediterranean area: What can be learned from a retrospective review analysis of the situation in Morocco during 1990 to 2010? Microorganisms, 8, 1511. 33008038 10.3390/microorganisms8101511 PMC7650785

[18] Kahime K, Boussaa S, El Mzabi A, Boumezzough A. 2015. Spatial relations among environmental factors and phlebotomine sand fly populations (Diptera: Psychodidae) in central and southern Morocco. Journal of Vector Ecology, 40, 342–354. 26611970 10.1111/jvec.12173

[19] Kahime K, Boussaa S, Idrissi AL-E, Nhammi H, Boumezzough A. 2016. Epidemiological study on acute cutaneous leishmaniasis in Morocco. Journal of Acute Disease, 5, 41–45.

[20] Kahime K, Boussaa S, Nhammi H, Boumezzough A. 2017. Urbanization of human visceral leishmaniasis in Morocco. Parasite Epidemiology and Control, 2, 1–6. 10.1016/j.parepi.2017.07.001 PMC5952660 29774290

[21] Kahime K, Sereno D, Bounoua L, El Hidan MA, Bouhout S. 2018. Management of leishmaniases in the era of climate change in Morocco. International Journal of Environmental Research and Public Health, 15, 1542.30037049

[22] Karmaoui A, Ben Salem A, Sereno D, El Jaafari S, Hajji L. 2022. Geographic distribution of *Meriones shawi*, *Psammomys obesus*, and *Phlebotomus papatasi* the main reservoirs and principal vector of zoonotic cutaneous leishmaniasis in the Middle East and North Africa. Parasite Epidemiology and Control, 17, e00247. 35310083 10.1016/j.parepi.2022.e00247 PMC8931442

[23] Karmaoui A, El Qorchi F, Hajji L, Zerouali S. 2021. Eco-epidemiological aspects of zoonotic cutaneous leishmaniasis in Ouarzazate Province, Morocco. Journal of Parasitic Diseases, 45, 341–350. 34295032 10.1007/s12639-021-01368-z PMC8254845

[24] MATEE – Ministère de l’Aménagement du Territoire, de l’Eau et de l’Environnement. 2004. Stratégie nationale pour la conservation et l’utilisation durable de la diversité biologique. p. 120.

[25] Miller JM. 2010. Implications of the One Health paradigm for clinical microbiology. Clinical Microbiology Newsletter, 32, 51–56. 32287681 10.1016/j.clinmicnews.2010.03.003 PMC7115297

[26] Ministère de la Santé et de la Protection sociale, Maroc. 2016. Santé en chiffres 2015.

[27] Mouloudi I, Zitouni N, Himi H, Injai U, Toumi A, Rahhou I, Legssyer B. 2024. Epidemiological features of leishmaniasis and implications for public health in the eastern region of Morocco. E3S Web of Conferences, 527, 02016.

[28] Oliveira R, Pimentel KBA, Moura MES, Pinheiro VCS. 2023. Impacto da COVID-19 no registro de casos de leishmaniose tegumentar no Maranhão, Brasil. Revista de Epidemiologia e Controle de Infecção, 13(3). 10.17058/reci.v13i3.18352.

[29] Organisation des Nations Unies pour l’Alimentation et l’Agriculture. 2014. Deuxième rapport national sur l’état des ressources génétiques animales. p. 46.

[30] Organisation des Nations Unies pour l’Alimentation et l’Agriculture. 2025. Observation de la Terre. FAO. 20258191

[31] Organisation Mondiale de la Santé. 2016. La leishmaniose dans les pays à forte charge de morbidité: mise à jour épidémiologique à partir des données notifiées en 2014.

[32] Paranaiba LF, Pinheiro LJ, Torrecilhas AC, Macedo DH, Menezes-Neto A, Tafuri WL, Soares RP. 2017. *Leishmania enriettii* (Muniz & Medina, 1948): A highly diverse parasite is here to stay. PLoS Pathogens, 13, e1006303. 28542526 10.1371/journal.ppat.1006303 PMC5444841

[33] Rhajaoui M. 2011. Les leishmanioses humaines au Maroc : une diversité nosogéographique. Pathologie-Biologie, 59, 226–229. 19942371 10.1016/j.patbio.2009.09.003

[34] Rioux JA, Lanotte G, Les leishmanioses cutanées du bassin Méditerranéen occidental. De l'identification enzymatique à l'analyse éco-épidémiologique. L'exemple de 3 «foyers», tunisien, marocain et français, in Leishmania. Rioux JA, editor. Taxonomie et phylogénèse. (Coll. int. CNRS / INSERM, 1984). IMEEE: Montpellier; 196 p., pp. 365–395.

[35] Sereno D. 2026. When discovery meets neglect: rethinking disease emergence in cutaneous leishmaniasis and other neglected tropical diseases. Frontiers in Tropical Diseases, 6, 1744659.

[36] Uwishema O, Sapkota S, Wellington J, Onyeaka CVP, Onyeaka H. 2022. Leishmaniasis control in the light of the COVID-19 pandemic in Africa. Annals of Medicine and Surgery, 80, 104263. 35936565 10.1016/j.amsu.2022.104263 PMC9339101

